# Precision switching from entecavir to tenofovir alafenamide in chronic hepatitis B: a narrative review of matching the patient phenotype to efficacy, safety, and value

**DOI:** 10.12701/jyms.2026.43.55

**Published:** 2026-08-10

**Authors:** Soon Woo Nam

**Affiliations:** Hepatobiliary Unit, Division of Gastroenterology, Department of Internal Medicine, Incheon St. Mary’s Hospital, College of Medicine, The Catholic University of Korea, Seoul, Korea

**Keywords:** Drug substitution, Entecavir, Chronic hepatitis B, Precision medicine, Tenofovir

## Abstract

Switching patients who are virologically stable with chronic hepatitis B from entecavir to tenofovir alafenamide has become routine in Korea; however, the literature has established that switching works at the population level, but not at the patient level. This review reframes this decision as one of candidate selection. Integrating a 2026 Korean multicenter randomized trial with 2023 to 2025 real-world and pharmacoeconomic data shows that in patients who are fully suppressed, the switch is non-inferior and safe but adds little, and the analysis separates evidence generated against tenofovir disoproxil fumarate from the sparser entecavir-controlled evidence on which switching decisions must rest. Five phenotypes with a favorable benefit–risk profile are then defined—low-level viremia or partial virologic response, declining estimated glomerular filtration rate or early tubular injury, bone loss or fracture risk, entecavir or multidrug resistance, and planned or confirmed pregnancy—alongside two conditional situations: decompensated cirrhosis, where routine switching is unnecessary unless renal or bone disease coexists, and high cardiovascular risk, which requires lipid monitoring rather than exclusion. Each phenotype is mapped to the 2026 Korean Association for the Study of the Liver guideline and to Korean reimbursement criteria revised in November 2025, graded for certainty, and consolidated into one algorithm that transfers across health systems because the phenotypes are internationally recognized.

## Introduction

Durable viral suppression with a high barrier to resistance is the central objective in the long-term management of chronic hepatitis B (CHB), because it governs the progression to cirrhosis and hepatocellular carcinoma (HCC) [1]. Entecavir (ETV), tenofovir disoproxil fumarate (TDF), and tenofovir alafenamide (TAF) are the recommended first-line nucleos(t)ide analogs [1]. TAF, a hepatocyte-targeted tenofovir prodrug, achieves higher intrahepatic tenofovir diphosphate concentrations while lowering systemic tenofovir exposure by more than 90%, which mitigates the renal and skeletal liabilities associated with TDF [2]. Against this pharmacological backdrop, ETV recipients who are virologically stable are increasingly moved to TAF in routine practice; however, the switch is often applied indiscriminately, as though the agent, rather than the patient, is the unit of decision.

This review aims to reverse this logic. Rather than asking whether TAF is a reasonable drug, it asks, at the individual patient level, who genuinely benefits from being switched on the grounds of antiviral efficacy, drug specificity, safety, and cost, and how that decision aligns with clinical practice guidelines and reimbursement rules. The intended readership is practicing hepatologists, who must decide whether a change in agent is warranted for a specific patient who is ETV-treated and stable.

For this synthesis, PubMed, Embase, and the Cochrane Library were searched (January 2015–June 2026) by combining the terms “chronic hepatitis B,” “entecavir,” “tenofovir alafenamide,” “switch,” “low-level viremia,” “renal,” “bone mineral density,” “drug resistance,” “pregnancy,” “decompensated cirrhosis,” and “HBsAg,” with priority given to randomized trials, comparative cohorts, and meta-analyses, alongside current guidance from the Korean Association for the Study of the Liver (KASL), the European Association for the Study of the Liver (EASL), and the American Association for the Study of Liver Diseases (AASLD). Korean reimbursement criteria were drawn from the Ministry of Health and Welfare (MOHW) notice on detailed criteria for pharmaceutical benefits and from the Health Insurance Review and Assessment Service (HIRA) guidance, as revised on November 1, 2025. This is a framework-oriented narrative synthesis where evidence rests on small or single-center studies, which is stated explicitly, and the certainty of each recommendation is graded qualitatively.

## What this review adds

The ETV-to-TAF switch is not a new topic, and any further review must justify itself against the mature literature. Four reference points define the field, and the present study departs from each in a specific manner (Table 1).

The 2022 narrative review by Lim et al. [2] competently summarized the case for switching patients with CHB to TAF, but its evidence base predates both the pivotal 2026 Korean randomized trial [3] and the 2024 to 2025 real-world low-level viremia (LLV) and renal recovery cohorts. More fundamentally, it reasons at the level of the drug class (“is TAF a reasonable choice?”) rather than at the level of the patient (“which ETV-treated patient should be moved, and which should not?”) and offers no selection algorithm, explicit boundary conditions, or payer mapping.

The 2024 systematic review and meta-analysis by Shahzil et al. [4] is the strongest quantitative synthesis to date, showing that switching from ETV to tenofovir increases the odds of a complete virologic response roughly 5.6-fold and deepens hepatitis B virus (HBV) DNA suppression by approximately 1.03 log_10_ IU/mL. By design, its output is a population-averaged effect estimate that does not resolve heterogeneity in clinically actionable subgroups, does not weigh the countervailing lipid signal, and cannot tell a clinician facing a specific patient whether the expected benefit justifies the switch. The contribution here is the translational step, converting pooled efficacy into phenotype-level indications and boundaries.

A widely cited single-center switching cohort [5] quantified hepatitis B surface antigen (HBsAg) kinetics after switching (adjusted hazard ratio, 2.75 for a 90% HBsAg decline in the ETV→TAF group; n=286) but is a single-outcome dataset rather than a decision framework. It is incorporated here, together with a contrasting long-term cohort in which HBsAg decreased only in patients with a low baseline antigen level, to calibrate realistic expectations for the serologic phenotype rather than to overstate the functional cure potential. Finally, the comparative safety and pharmacoeconomic literature, a network meta-analysis ranking the agents for renal and skeletal safety [6] and cost-effectiveness models built around single scenarios [7], addresses safety and value as separate questions; the present review folds both into one selection logic.

In short, prior work answers whether switching helps on average; the present review operationalizes for whom, against what boundaries, with what certainty, and within which guideline and reimbursement pathway, consolidated into a single decision algorithm (Fig. 1). To our knowledge, this is the first synthesis to incorporate the 2026 Korean randomized trial and couple the clinical decision explicitly to the Korean payer criteria, as revised in November 2025.

## Pharmacologic rationale and antiviral efficacy

The plasma stability and preferential hepatocyte delivery of TAF yield yield high intracellular tenofovir diphosphate concentrations, which is the basis for both its potency in patients who are imperfectly suppressed and its renal and skeletal advantages over TDF [2]. In a pivotal Korean phase IV trial (13 hospitals; 196 patients; ClinicalTrials.gov NCT06000657), patients suppressed on ETV who switched to TAF (n=95) vs. those who continued ETV (n=101) had week-48 per-protocol HBV DNA suppression of 100.0% vs. 99.0% (non-inferior), with comparable renal and lipid changes (Table 2) [3]. Therefore, the switch carries no virologic penalty in a patient who is stable, but it also confers little incremental benefit, which is why selection matters.

Two evidence streams should be separated. The renal and skeletal advantage of TAF was established principally against TDF, the comparator in the registrational and switch programs, and followed from the reduction in systemic tenofovir exposure [2]. ETV is not TDF; in a systematic review and meta-analysis of 16 comparative studies, TDF was more harmful to renal function than ETV within the first 24 months, whereas serum creatinine did not differ significantly between the two agents and no significant difference in bone mineral density (BMD) was detected between them [8]. Therefore, ETV is a comparatively benign comparator for renal and skeletal endpoints. Indeed, in the network meta-analysis, the only significant pairwise comparison between TAF and ETV runs in ETV’s favor, where the effect on creatinine rise was greater with TAF (standardized mean difference, −0.55; 95% confidence interval [CI], −1.01 to −0.09), and ETV ranked the best of the three agents on that outcome [6]. The incremental organ-safety gain of moving from ETV, as distinct from moving from TDF, to TAF is correspondingly smaller, rests on fewer and smaller studies, and was not demonstrable in the unselected patients of the Korean randomized trial who were suppressed, in whom the week-48 change in estimated glomerular filtration rate (eGFR) was −4.0±10.8 mL/min/1.73 m^2^ with TAF and −3.5±12.3 mL/min/1.73 m^2^ with ETV (*p*=0.44) (Table 2) [3]. Accordingly, TDF-controlled data are used below to describe the pharmacological properties of TAF, whereas recommendations on who should switch from ETV are graded on ETV-controlled evidence alone.

## A precision framework for candidate selection

Fig. 1 summarizes the proposed pathway. The patient is profiled across five axes, each defining a phenotype in which the incremental value of TAF is supported by phenotype-specific data and can be matched to a reimbursement route (Table 3). Serologic response is treated as a secondary consideration rather than as a sixth axis because the supporting evidence is subgroup-confined. Situations that modify rather than forbid the decision (i.e., decompensated cirrhosis and high cardiovascular risk) are considered separately under the section “Boundaries: conditional rather than absolute.”

### 1. Low-level viremia and partial virologic response

Persistent LLV sustains mutational pressure and necroinflammation, increasing the risks of resistance, fibrosis, and HCC [4]. In a prospective 48-week real-world study of 351 patients with LLV who were ETV-treated, switching to TAF achieved a complete virological response in 75.3% vs. 11.4% of patients with continued ETV (*p*<0.001), and alanine aminotransferase (ALT) normalized in 67.8% vs. 17.1% of them, respectively (*p*<0.001) [9]. A 2-year paired cohort study found undetectable HBV DNA maintenance rising from 33.3% during the ETV period to 68.9% after switching (*p*<0.01) [10], and a single-center randomized suboptimal-response study indicated a week-48 virologic response of 93.3% vs. 66.7% (*p*=0.012) with a greater HBV DNA decline (−3.8 log_10_ IU/mL vs. −2.4 log_10_ IU/mL, *p*<0.001) [11]. This phenotype showed the largest increase in efficacy (certainty: moderate).

### 2. Declining estimated glomerular filtration rate and early tubular injury

ETV is eliminated renally, and long-term nucleos(t)ide exposure can contribute to subtle glomerular dysfunction. In a network meta-analysis of long-term nucleos(t)ide safety, TAF received the most favorable ranking of the three agents for eGFR preservation [6]. Ranking statistics order agents rather than establish a significant difference between any two of them, and in a pairwise meta-analysis of 16 comparative studies, TDF and not ETV was more harmful to renal function, the disadvantage being confined to the first 24 months [8]. The only significant TAF-versus-ETV comparison in the network meta-analysis ran in the opposite direction, ETV being the least likely of the three agents to raise serum creatinine levels [6]. Therefore, direct evidence of renal gain from an ETV-to-TAF switch is confined to a very small and highly selected group. In a propensity-matched retrospective cohort of 179 patients, the six patients moved from ETV to TAF because of an emergent eGFR abnormality at week 48 recovered eGFR from 83.6±5.5 to 93.4±9.9 mL/min/1.73 m^2^ (*p*=0.031), with a concomitant fall in serum creatinine level (*p*=0.046) between weeks 48 and 60; with only six patients, no concurrent unswitched control treated for the same indication, and a retrospective design, regression to the mean cannot be excluded [12]. In unselected patients with preserved renal function who were virologically suppressed, a randomized trial found no eGFR advantage at week 48 (Table 2) [3]. Thus, the candidate is a patient with a documented, ongoing decline in eGFR or with tubular injury, and not a patient whose renal function is stable. A practical consideration reinforces the pharmacologic one: below a creatinine clearance of 50 mL/min, ETV requires interval extension or dose reduction, whereas TAF is administered as a single unadjusted 25-mg dose down to an eGFR of 15 mL/min/1.73 m^2^ [13,14] (certainty: low).

### 3. Bone loss and fracture risk

A network meta-analysis of long-term nucleos(t)ide safety ranked TAF as the least likely to cause BMD loss (surface under the cumulative ranking curve, 19.6% vs. 50.6% and 79.7% for ETV and TDF, respectively) [6]. As with renal endpoints, this is a ranking rather than a demonstrated TAF-versus-ETV difference; the only significant bone comparison in that analysis was TAF vs. TDF [6], and the pairwise meta-analysis found no significant difference in BMD, even between TDF and ETV [8], which puts the expected magnitude of any TAF-versus-ETV difference in perspective. The only randomized ETV-controlled BMD data come from a single-center trial of 60 patients with a suboptimal response to ETV, in which switching to TAF limited the week-48 BMD loss at the spine and hip to −0.8% and −0.6%, respectively, vs. −2.1% and −1.8%, respectively, with continued ETV use (*p*=0.004 and *p*=0.007, respectively) [11]; the endpoint was densitometric rather than fracture-based, follow-up was 1 year, and the population was selected for incomplete virologic suppression, so the estimate should not be extrapolated to patients who are fully suppressed. Patients with established osteoporosis or a prior fragility fracture, the group for which Korean reimbursement is available, are reasonable candidates. Osteopenia alone is a weaker indication; no switching study has shown fracture outcomes, and the decision should rest on absolute fracture risk and whether other drugs or comorbidities contribute to bone loss rather than on the BMD trajectory alone (certainty: low).

### 4. Entecavir or multidrug resistance

Confirmed genotypic resistance is the least contentious reason for replacing ETV, but the evidence supporting TAF specifically deserves to be stated precisely. ETV has a low genetic barrier in patients who are lamivudine-experienced; therefore, rescue requires a non-cross-resistant nucleotide, and classical evidence for this principle was generated with TDF rather than with TAF. Direct TAF data were obtained from patients already established on a tenofovir backbone. In a multicenter randomized non-inferiority trial of 174 patients with HBV resistant to lamivudine, ETV, and/or adefovir who had received TDF monotherapy for at least 96 weeks, switching to TAF (n=87) maintained HBV DNA <60 IU/mL in 98.9% of patients at week 48 vs. 97.7% with continued TDF (difference, 1.1%; 95% CI, −2.7% to 5.0%), with a greater median reduction in ALT levels (−3 IU/L vs. +2 IU/L, *p*=0.02) [15], and suppression was maintained through 3 years of subsequent follow-up [16]. These data establish TAF as a durable option for drug-resistant HBV once suppression has been achieved. However, they do not measure the outcome of moving a patient who is viremic and ETV-resistant directly from ETV to TAF, for which, to our knowledge, no randomized comparison exists. The practical recommendation is, therefore, unchanged; confirmed ETV or multidrug resistance warrants a change to a tenofovir prodrug, and Korean reimbursement covers TAF for this indication, alone or in combination, whereas the certainty attached to TAF rather than TDF as the destination remains inferential (certainty: low–moderate).

### 5. Planned or confirmed pregnancy

Pregnancy is a reason to move a woman from ETV to a tenofovir prodrug and not a reason to withhold TAF. ETV is not the preferred agent for women of childbearing potential who conceive or intend to conceive, whereas both tenofovir prodrugs now have supportive evidence. In a systematic review and network meta-analysis of 31 studies (2,588 mothers who were highly viremic and receiving TDF, 280 receiving TAF, and 1,600 untreated), maternal TDF prophylaxis reduced mother-to-child transmission (MTCT) in infants aged 6 to 12 months (risk ratio, 0.10; 95% CI, 0.07–0.16), while pairwise and network comparisons showed TAF and TDF to be equally effective (risk ratio, 1.09; 95% CI, 0.15–7.65), with no safety concerns for mothers or infants in any regimen [17]. Accordingly, the 2026 KASL guideline lists TAF together with TDF among the agents appropriate for women who are pregnant or preparing for pregnancy [18], and, following the MOHW notice effective November 1, 2025, Korean reimbursement now covers TAF (as it does TDF) for the prevention of vertical transmission in mothers with HBV DNA ≥200,000 IU/mL [19]. Either prodrug is, therefore, an acceptable destination for women who are ETV-treated. TDF retains a larger cumulative human pregnancy exposure, whereas TAF is the more logical choice where renal or skeletal comorbidity coexists (certainty: moderate).

## A secondary consideration: serologic response

Serological gains should not be overstated, and is due to differences in study populations rather than a true treatment effect. In the suboptimal-response trial, hepatitis B e antigen (HBeAg) seroconversion was more frequent with TAF (33.3% vs. 8.3%, *p*=0.04) [11] because residual replication can allow an immune-mediated response; only 12 patients per arm were HBeAg-positive at baseline. However, the comparison rests on four patients vs. one, and the trial’s own abstract lists the serologic endpoints as not differing between the groups. In the patients in the trial who were fully suppressed and predominantly HBeAg negative, no such advantage was observed, and the week-48 difference (0.0% vs. 12.5%, *p*=0.21) likewise rests on very few eligible patients and should not be read as favoring either agent (Table 2) [3]. Neither result is a reliable basis for expecting a serological gain from switching. For HBsAg, the pattern is the same: a single-center cohort linked ETV→TAF exposure to a faster 90% HBsAg decline (adjusted hazard ratio, 2.75; n=286) [5], whereas a long-term study found no significant change across the whole switched cohort, the significant decline and the three observed HBsAg losses being confined to patients with a baseline HBsAg <100 IU/mL [20]. Young patients pursuing a functional cure who are lean and have low antigen levels may therefore be considered, but with realistic expectations of small absolute gains (certainty: low).

## Guidelines and reimbursement alignment

Korean reimbursement grants continue coverage after a patient is switched from ETV to TAF on a case-by-case basis under three long-standing routes [19]: confirmed drug resistance (including multidrug resistance); a documented severe adverse effect (osteoporosis [dual-energy X-ray absorptiometry T-score ≤−2.5 or a radiographically confirmed osteoporotic fracture] or eGFR <60 mL/min/1.73 m^2^ [renal proteinuria adjudicated individually]); or partial virologic response after at least 12 months on a high-genetic-barrier agent, switched to a non-cross-resistant agent (a TDF-to-TAF change for this indication is not reimbursed). The MOHW notice effective November 1, 2025, substantially widened the remaining boundaries. TAF is now reimbursed as first-line therapy in patients who are HBV DNA-positive with decompensated cirrhosis or HCC (without the previous restriction to those with renal impairment or osteoporosis), after liver transplantation, as prophylaxis against reactivation, and for MTCT prophylaxis in mothers with HBV DNA ≥200,000 IU/mL [19]. These rules run alongside the 2026 KASL guidelines, which grade the natural history of CHB by HBV DNA rather than by ALT; recommend ETV, TDF, TAF, and besifovir as first-line agents; endorse changing to tenofovir when partial virologic response persists on ETV; and name TAF among the preferred options in renal disease, bone and metabolic disease, and pregnancy [18]. Table 3 maps each phenotype to its rationale, reimbursement route, and evidence certainty. Although these criteria are Korean, the underlying phenotypes (i.e., resistance, renal, bone, incomplete suppression, and pregnancy) are recognized by the EASL and AASLD; therefore, the framework is readily transferred to other payer systems.

## Boundaries: conditional rather than absolute

### 1. Lipid changes and cardiovascular risk

The most consistent adverse consequence of switching is an increase in serum lipid levels. In a propensity-matched cohort of 336 patients (TAF, n=214; ETV, n=122), 1-year total cholesterol was higher with TAF than with ETV (180.6 vs. 168.6 mg/dL [4.67 vs. 4.36 mmol/L], *p*=0.006). Within the matched model, TAF raised total cholesterol by approximately 5% above baseline (*p*=0.019); triglycerides also rose from baseline in the TAF group (*p*=0.014), whereas ETV produced no significant change in any lipid fraction, and the incidence of fatty liver did not rise over 1 year [21]. The randomized trial did not reproduce a significant between-group difference over 48 weeks (change in total cholesterol, 4.9±27.5 vs. 2.0±26.1 mg/dL; *p*=0.41) (Table 2) [3]; the direction of change was the same, but that trial enrolled patients who were fully suppressed, followed them for a shorter period, and was powered for virologic non-inferiority rather than for lipid endpoints, so the observational estimate is best read as the upper bound of what to expect. This signal reflects loss of the systemic tenofovir lipid-lowering effect [22]. Therefore, patients with preexisting dyslipidemia or high cardiovascular risk warrant lipid monitoring and, where indicated, statin therapy, rather than exclusion from switching (certainty: moderate).

### 2. Decompensated cirrhosis (Child-Turcotte-Pugh class B or C)

TAF 25 mg is licensed only for compensated liver disease, and the registration program excluded decompensation [13]. However, this labeling limitation is no longer matched by clinical experience. In patients who are treatment naive and presenting with first decompensation, 48 weeks of TAF produced a complete virologic response in 90.7% of them and a ≥2-point improvement in the Child-Turcotte-Pugh score in 79.1%, with no significant change in serum creatinine level, eGFR, or total cholesterol level, although cystatin C and low-density lipoprotein cholesterol levels rose [23]. In a propensity-matched comparison in HBV-related decompensated cirrhosis, eGFR was unchanged with TAF (remained at 101.9 mL/min/1.73 m^2^) but declined with TDF (103.6 to 99.1 mL/min/1.73 m^2^), and a ≥25% fall in eGFR occurred in 5.2% vs. 10.5% of patients, respectively [24]. Korean policy has changed accordingly; since November 1, 2025, ETV, TDF, and TAF (but not besifovir) have all been reimbursed as first-line therapies in decompensated cirrhosis, with the earlier restriction of TAF to patients with renal impairment or osteoporosis removed [19].

The implications for switching are specific, rather than prohibitive. ETV retains the largest body of long-term outcome data in decompensated disease [25], and (rare ETV-associated lactic acidosis notwithstanding [26]) remains a sound choice; in a patient who is stable on it, replacement achieves nothing, and routine ETV-to-TAF switching is not indicated. A patient who is decompensated and develops renal dysfunction or established bone disease on ETV is a patient for whom the hepatocyte-targeted prodrug was designed. Such patients should be regarded as appropriate candidates rather than categorically excluded. Because the supporting data are retrospective and modest in size and because lipid and cystatin C increases have been reported in this population, switching warrants closer monitoring of hepatic function, renal function, and lipid levels than in compensated disease (certainty: low). Emerging evidence that TAF may improve outcomes in HBV-related acute-on-chronic liver failure [27] requires confirmation before practice changes.

## Drug specificity and value

Value considerations sharpen the framework. ETV is available as inexpensive generics, so switching a patient who is stable and low risk to branded TAF adds cost without measurable return; formal analyses support continuing the less expensive agent in such patients [7]. The economic case for TAF is subgroup-specific and relies on averted downstream costs: reduced HCC and cirrhosis in patients with LLV [28], avoidance of dose-adjustment complexity and renal replacement in patients with renal impairment, fracture avoidance in patients with bone disease, and averted chronic infection in infants when vertical transmission is prevented. Hepatocyte-targeted specificity and a single unadjusted daily dose are factors that make TAF worth the incremental price in these patients and unnecessary in the stable majority.

## Certainty of evidence, limitations, and research gaps

Several recommendations rest on small, single-center, or short-duration studies, and the pivotal randomized data derived from a 2026 trial are only now entering the literature; effect sizes may prove optimistic, and the serologic benefits are modest and subgroup-confined. Much of the renal and skeletal reasoning for TAF was generated in TDF-controlled studies and is only indirectly applicable to patients treated with ETV; the ETV-controlled data are few, small, and largely restricted to patients selected for incomplete suppression or emergent organ dysfunction, which is why the renal and bone phenotypes are graded low. The decompensated cirrhosis evidence is entirely retrospective, the resistance evidence for TAF derives from patients who are TDF-experienced rather than those who are ETV-treated, and the pregnancy evidence for TAF, although consistent, rests on far fewer exposures than for TDF. Priorities for future work include adequately powered randomized trials of switching in the LLV phenotype with hard endpoints (e.g., HCC and fibrosis progression) rather than surrogate virologic response alone; head-to-head renal and bone comparisons of TAF vs. ETV, rather than TAF vs. TDF, in the switching population, with fracture and tubular endpoints; long-term cardiovascular follow-up quantifying the clinical impact of post-switch lipid increases; prospective evaluation of switching within decompensated cirrhosis, with hepatic, renal, and metabolic endpoints; longer-term infant follow-up after in utero TAF exposure; and phenotype-stratified cost-effectiveness modeling across health systems.

## Conclusion

The value of switching from ETV to TAF lies not in whether it can be done, but in choosing which patient to switch. In patients who are fully suppressed, the switch is non-inferior, safe, and largely unnecessary. Priority candidates are those with LLV or partial virologic response, a documented decline in eGFR or tubular injury, bone loss or fracture risk, ETV or multidrug resistance, or a planned or confirmed pregnancy, with younger patients who are lean and have low antigen levels as a secondary consideration for accelerating a functional cure [3,6,9,11,12,15-17]. Because much of the renal and skeletal evidence for TAF was generated against TDF, the strength of the recommendation differs by phenotype; it is the strongest for LLV, resistance, and pregnancy and weakest (graded low) for renal and bone phenotypes, where the ETV-controlled data are small and confined to selected patients. The remaining boundaries are conditional rather than absolute. In decompensated cirrhosis, routine switching adds nothing to ETV, yet concomitant renal dysfunction or bone disease makes the switch appropriate under closer monitoring, and all three first-line agents are now reimbursed in this setting in Korea [19,23-25]. In addition, the post-switch lipid rise calls for monitoring and, where indicated, statin therapy rather than exclusion [21]. By applying the phenotype- and value-aligned algorithms proposed here, the ETV-to-TAF switch becomes a high-value guideline-concordant precision strategy.

## Figures and Tables

**Fig. 1.**
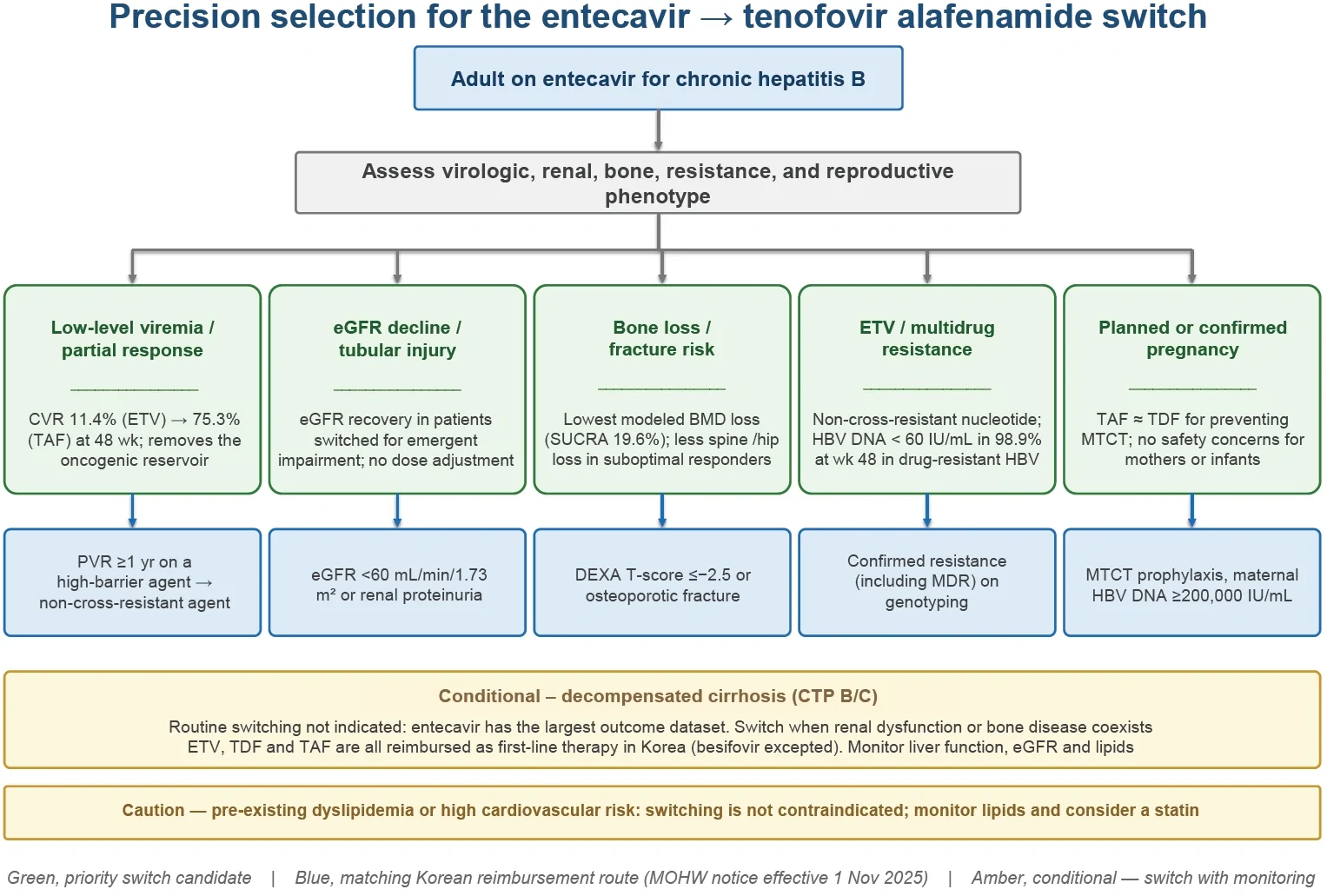
Phenotype-based algorithm for selecting entecavir-to-tenofovir alafenamide switch candidates, with matched Korean reimbursement routes. Green, priority switch candidate; blue, matching Korean reimbursement route (Ministry of Health and Welfare notice effective November 1, 2025); amber, conditional-switch with monitoring. ETV, entecavir; eGFR, estimated glomerular filtration rate; TAF, tenofovir alafenamide; CVR, complete virologic response; BMD, bone mineral density; SUCRA, surface under the cumulative ranking curve; HBV, hepatitis B virus; TDF, tenofovir disoproxil fumarate; MTCT, mother-to-child transmission; PVR, partial virologic response; DEXA, dual-energy X-ray absorptiometry; MDR, multidrug resistance; MOHW, Ministry of Health and Welfare; CTP, Child-Turcotte-Pugh. Serologic response is discussed in the text as a secondary consideration and is not shown as a switching axis.

**Table 1.** Positioning of the present review relative to prior literature

Attribute	Lim et al. 2022 [2]	Shahzil et al. 2024 [4]	Single-center 2021 [5]	Current review
Study design type	Narrative review	Systematic review and meta-analysis	Cohort study	Narrative review
Unit of analysis	Drug class	Drug class (pooled)	One outcome (HBsAg)	Patient phenotype
Primary output	Narrative summary	Pooled effect sizes	HBsAg kinetics	Selection algorithm (Fig. 1)
2026 Korean RCT	No	No	No	Yes
Safety subgroups	Partial	No	No	Renal, bone, lipid
Boundary definition	Partial	No	No	Explicit, including conditional
Guideline + payer map	No	No	No	Yes (2026 KASL + 2025 MOHW)
Value/cost framing	Limited	No	No	Phenotype-specific
Evidence grading	No	GRADE (pooled)	No	Per-phenotype (Table 3)

HBsAg, hepatitis B surface antigen; RCT, randomized controlled trial; KASL, Korean Association for the Study of the Liver; MOHW, Ministry of Health and Welfare; GRADE, Grading of Recommendations Assessment, Development and Evaluation. Data from references [2–7] and the present synthesis.

**Table 2.** Week-48 outcomes in the Korean multicenter switch trial

Endpoint (week 48)	TAF switch (n=95)	ETV maintain (n=101)	*p*-value
HBV DNA suppression, PPS	100.0%	99.0%	Non-inferior
ALT normalization (AASLD)	55.0%	38.7%	0.26
HBeAg seroconversion	0.0%	12.5%	0.21
Δ eGFR (mL/min/1.73 m^2^)	−4.0±10.8	−3.5±12.3	0.44
Δ Total cholesterol (mg/dL)	+4.9±27.5	+2.0±26.1	0.41
Δ LDL cholesterol (mg/dL)	+2.4±20.6	+1.1±25.9	0.29
Δ Triglycerides (mg/dL)	+20.5±119.9	+6.0±65.1	0.46

Values are presented as percentage or mean±standard deviation. TAF, tenofovir alafenamide; ETV, entecavir; HBV, hepatitis B virus; PPS, per-protocol set; ALT, alanine aminotransferase; AASLD, American Association for the Study of Liver Diseases; HBeAg, hepatitis B e antigen; eGFR, estimated glomerular filtration rate; LDL, low-density lipoprotein; Δ, mean change from baseline. Between-group lipid and eGFR changes were not statistically significant. Data from reference [3]. HBeAg seroconversion was assessed only in the small number of HBeAg-positive participants and did not differ significantly; the opposite direction reported in patients with a suboptimal response to entecavir (reference [11]) reflects a different population rather than a conflicting effect.

**Table 3.** Switch candidates, conditional situations, reimbursement matching, and evidence certainty

Phenotype	Clinical rationale (key data)	Korean reimbursement route (MOHW)	Certainty
Priority candidate: low-level viremia / partial virologic response	CVR 11.4% (ETV) vs. 75.3% (TAF) at 48 wk; removes a persistent oncogenic reservoir [9]	Partial virologic response after ≥1 yr on a high-barrier agent → switch to a non-cross-resistant agent	Moderate
Priority candidate: declining eGFR / early tubular injury	eGFR recovery reported in only six patients switched for emergent impairment [12]; no advantage in unselected suppressed patients [3]; TAF ranks first for eGFR preservation, but the only significant TAF-versus-ETV comparison (creatinine) favors ETV [6,8]	eGFR <60 mL/min/1.73 m^2^ or renal proteinuria (case-by-case)	Low
Priority candidate: bone loss/fracture risk	Lowest modeled probability of BMD decline (SUCRA 19.6%) [6]; less spine/hip loss than ETV in one small randomized trial of suboptimal responders, without fracture data [11]	DEXA T-score ≤−2.5 or radiographic osteoporotic fracture	Low
Priority candidate: ETV or multidrug resistance	Tenofovir prodrug required for non-cross-resistance; in drug-resistant HBV, TAF maintained HBV DNA <60 IU/mL in 98.9% at wk 48 (non-inferior to TDF) and through 3 yr [15,16]; no randomized ETV→TAF comparison	Confirmed resistance (incl. MDR) on genotyping → TAF, alone or in combination	Low–moderate
Priority candidate: planned or confirmed pregnancy	TAF and TDF equally effective for MTCT prevention, with no safety concerns for mothers or infants [17]; TAF listed for pregnancy in the 2026 KASL guideline [18]	MTCT prophylaxis in mothers with HBV DNA ≥200,000 IU/mL (since 1 Nov 2025)	Moderate
Conditional: decompensated cirrhosis (CTP B/C)	Routine switching unnecessary—ETV has the largest outcome dataset [25]; but TAF achieves CVR 90.7% and ≥2-point CTP improvement in 79.1% [23] and preserves eGFR better than TDF [24]. Switch when renal dysfunction or bone disease coexists; monitor liver function, renal function, and lipids	ETV, TDF, and TAF all reimbursed as first-line therapy (besifovir excepted), unrestricted since 1 Nov 2025	Low
Caution: high cardiovascular risk/dyslipidemia	Total cholesterol and triglycerides rise after switching [21]; not a contraindication	No separate route; switch justified by another phenotype	Moderate

MOHW, Ministry of Health and Welfare; CVR, complete virologic response; ETV, entecavir; TAF, tenofovir alafenamide; eGFR, estimated glomerular filtration rate; BMD, bone mineral density; SUCRA, surface under the cumulative ranking curve; DEXA, dual-energy X-ray absorptiometry; HBV, hepatitis B virus; TDF, tenofovir disoproxil fumarate; MDR, multidrug resistance; MTCT, mother-to-child transmission; KASL, Korean Association for the Study of the Liver; CTP, Child-Turcotte-Pugh. Serologic response is discussed in the text as a secondary consideration and is not listed here as a switch phenotype.

## References

[1] European Association for the Study of the Liver (2017). EASL 2017 Clinical Practice Guidelines on the management of hepatitis B virus infection. J Hepatol.

[2] Lim YS, Seto WK, Kurosaki M, Fung S, Kao JH, Hou J (2022). Review article: switching patients with chronic hepatitis B to tenofovir alafenamide-a review of current data. Aliment Pharmacol Ther.

[3] Shin H, Hur MH, Baek YH, Lee CK, Cho YK, Lee JS (2026). Efficacy and safety of switching from entecavir to tenofovir alafenamide in chronic hepatitis B: a multicenter randomized trial in Korea. Gut Liver.

[4] Shahzil M, Chaudhary AJ, Kashif T, Qureshi AA, Muhammad A, Khan F (2024). Switching to tenofovir therapy versus continuation of entecavir for patients with hepatitis B virus infection: a systematic review and meta-analysis. JGH Open.

[5] Kumada T, Toyoda H, Yasuda S, Ito T, Tanaka J (2021). Impact of switching to tenofovir alafenamide fumarate in patients with entecavir-treated chronic hepatitis B. Eur J Gastroenterol Hepatol.

[6] Liu Z, Zhao Z, Ma X, Liu S, Xin Y (2023). Renal and bone side effects of long-term use of entecavir, tenofovir disoproxil fumarate, and tenofovir alafenamide fumarate in patients with hepatitis B: a network meta-analysis. BMC Gastroenterol.

[7] Sinakos E, Kachru N, Tsoulas C, Jeyakumar S, Smith NJ, Yehoshua A (2024). Cost-effectiveness of switching from tenofovir disoproxil fumarate to tenofovir alafenamide versus entecavir for chronic hepatitis B patients in Greece. J Comp Eff Res.

[8] Yang X, Yan H, Zhang X, Qin X, Guo P (2022). Comparison of renal safety and bone mineral density of tenofovir and entecavir in patients with chronic hepatitis B: a systematic review and meta-analysis. Int J Infect Dis.

[9] He MW, Cui L, Chen DD, Zhao Y, Luo WZ, Jia YF (2025). Efficacy and safety of switching from entecavir to tenofovir alafenamide in chronic hepatitis B patients with low-level viremia: a real-world 48-week extension study. Antimicrob Agents Chemother.

[10] Ishido S, Tamaki N, Uchihara N, Suzuki K, Tanaka Y, Miyamoto H (2023). Switching from entecavir to tenofovir alafenamide for maintaining complete virological response in chronic hepatitis B. JGH Open.

[11] Yuan GC, Chen AZ, Wang WX, Yi XL, Tu L, Peng F (2023). Efficacy and safety of tenofovir alafenamide in patients with chronic hepatitis B exhibiting suboptimal response to entecavir. World J Clin Cases.

[12] Wang L, Ma S, Malhi LR, Wu X, Liu L, Wan X (2025). Improvement in renal function after switching from entecavir to tenofovir alafenamide in chronic hepatitis B patients with low estimated glomerular filtration rates. Ann Hepatol.

[13] Gilead Sciences (2023). VEMLIDY^®^ (tenofovir alafenamide) prescribing information.

[14] Hepatitis B Online (c2026). Entecavir (Baraclude) [Internet]. https://www.hepatitisb.uw.edu/page/treatment/drugs/entecavir.

[15] Byun KS, Choi J, Kim JH, Lee YS, Lee HC, Kim YJ (2022). Tenofovir alafenamide for drug-resistant hepatitis B: a randomized trial for switching from tenofovir disoproxil fumarate. Clin Gastroenterol Hepatol.

[16] Choi J, Lim YS, Kim JH, Byun KS, Yoo BC, Multidrug-Resistant CHB Study Group (2023). Tenofovir alafenamide for multiple drug-resistant chronic hepatitis B: a 3-year clinical trial. Clin Gastroenterol Hepatol.

[17] Pan CQ, Zhu L, Yu AS, Zhao Y, Zhu B, Dai E (2024). Tenofovir alafenamide versus tenofovir disoproxil fumarate for preventing vertical transmission in chronic hepatitis B mothers: a systematic review and meta-analysis. Clin Infect Dis.

[18] Choi WM, Han JW, Kim TH, Choi M, Hur MH, Lee HY (2026). KASL clinical practice guidelines for management of chronic hepatitis B Endorsed by the East Asia Liver Alliance (EALA). Clin Mol Hepatol.

[19] Ministry of Health and Welfare (MOHW) (2025). Detailed criteria and methods for the application of health insurance benefits (pharmaceuticals): general principles for oral antivirals for chronic hepatitis B [Internet]. https://www.mohw.go.kr.

[20] Tajiri K, Hayashi Y, Murayama A, Muraishi N, Minemura M, Yasuda I (2024). Decrease in HBsAg after TAF switching from entecavir during long-term treatment of chronic hepatitis B virus infection. Viruses.

[21] Lai RM, Lin S, Wang MM, Li N, Zhou JH, Lin XY (2023). Tenofovir alafenamide significantly increased serum lipid levels compared with entecavir therapy in chronic hepatitis B virus patients. World J Hepatol.

[22] Suzuki K, Suda G, Yamamoto Y, Abiko S, Kinoshita K, Miyamoto S (2022). Effect of switching from tenofovir disoproxil fumarate to tenofovir alafenamide on lipid profiles in patients with hepatitis B. PLoS One.

[23] Rong X, Yang G, Xu Y, Chen H, Wang X, Fu J (2025). Efficacy and safety of tenofovir amibufenamide and tenofovir alafenamide for first-time HBV-related decompensated cirrhosis. J Viral Hepat.

[24] Chen L, Zhang HF, Cai YJ, Zhang L, Cai C, Lu MQ (2025). Comparative effectiveness and safety of tenofovir alafenamide versus tenofovir disoproxil fumarate in patients with HBV-associated decompensated cirrhosis: insights from a retrospective analysis. BMC Infect Dis.

[25] Park J, Jung KS, Lee HW, Kim BK, Kim SU, Kim DY (2017). Effects of entecavir and tenofovir on renal function in patients with hepatitis B virus-related compensated and decompensated cirrhosis. Gut Liver.

[26] Lange CM, Bojunga J, Hofmann WP, Wunder K, Mihm U, Zeuzem S (2009). Severe lactic acidosis during treatment of chronic hepatitis B with entecavir in patients with impaired liver function. Hepatology.

[27] Peng W, Gu H, Cheng D, Chen K, Wu C, Jiang C (2023). Tenofovir alafenamide versus entecavir for treating hepatitis B virus-related acute-on-chronic liver failure: real-world study. Front Microbiol.

[28] Sanai FM, Aljawad M, Alghamdi AS, Yehoshua A, Khathlan A, Alghamdi M (2024). Long-term health and economic benefits of switching to tenofovir alafenamide versus continuing on entecavir in chronic hepatitis B patients with low-level viremia in Saudi Arabia. Saudi J Gastroenterol.

